# Recent Advances in Micro- and Nano-Enhanced Intravascular Biosensors for Real-Time Monitoring, Early Disease Diagnosis, and Drug Therapy Monitoring

**DOI:** 10.3390/s25154855

**Published:** 2025-08-07

**Authors:** Sonia Kudłacik-Kramarczyk, Weronika Kieres, Alicja Przybyłowicz, Celina Ziejewska, Joanna Marczyk, Marcel Krzan

**Affiliations:** 1 Jerzy Haber Institute of Catalysis and Surface Chemistry, Polish Academy of Sciences, 8 Niezapominajek St., 30-239 Krakow, Poland; weronika.kieres@ikifp.edu.pl (W.K.); alicja.przybylowicz@student.pk.edu.pl (A.P.); marcel.krzan@ikifp.edu.pl (M.K.); 2 Faculty of Mechanical Engineering, Cracow University of Technology, 37 Jana Pawła II Av., 31-864 Krakow, Poland; joanna.marczyk@pk.edu.pl

**Keywords:** biosensor, nanotechnology, early disease detection, microtechnology, cardiovascular diagnostics, biomarker, continuous health monitoring, targeted drug delivery, personalized medicine, implantable diagnostic devices, quantum dots

## Abstract

Intravascular biosensors have become a crucial and novel class of devices in healthcare, enabling the constant real-time monitoring of essential physiological parameters directly within the circulatory system. Recent developments in micro- and nanotechnology have relevantly improved the sensitivity, miniaturization, and biocompatibility of these devices, thereby enabling their application in precision medicine. This review summarizes the latest advances in intravascular biosensor technologies, with a special focus on glucose and oxygen level monitoring, blood pressure and heart rate assessment, and early disease diagnostics, as well as modern approaches to drug therapy monitoring and delivery systems. Key challenges such as long-term biostability, signal accuracy, and regulatory approval processes are critical considerations. Innovative strategies, including biodegradable implants, nanomaterial-functionalized surfaces, and integration with artificial intelligence, are regarded as promising avenues to overcome current limitations. This review provides a comprehensive roadmap for upcoming research and the clinical translation of advanced intravascular biosensors with a strong emphasis on their transformative impact on personalized healthcare.

## 1. Introduction

A biosensor may be defined as an analytical instrument used to measure variations in biological activity—for instance, of enzymes, acids, or cells—and then transform them into quantifiable electronic signals [1]. The evolution of biosensor technologies has revolutionized healthcare in the 21st century, particularly in the field of real-time health monitoring. Nowadays, they are successfully applied in many fields, including medicine, pharmaceutics, industry, water quality management, and precision agriculture; for instance, they are used in diagnosing infections caused by bacterial and viral agents [2], detecting heavy metals [3], monitoring cholesterol levels [4], alerting personnel to water biotoxicity [5], detecting biomolecules [6], and enabling early cancer diagnosis [7]. According to the literature data, the market size of biosensors was estimated at USD 30.25 billion in 2024. Furthermore, biosensors are undoubtedly gaining increasing popularity, and this trend will continue, as indicated by the compound annual growth rate (CAGR) amounting to 8.7%, estimated from 2025 to 2034 [8]. The urgent need for reliable, real-time monitoring tools, particularly for chronic diseases, underscores the critical importance of innovations in intravascular biosensing.

Intravascular biosensors represent a groundbreaking achievement, as they bridge traditional diagnostic approaches with practical methods for the assessment of physiological parameters in patients. These devices are designed to operate within the human circulatory system, enabling unparalleled opportunities for the early detection and continuous monitoring of diseases; thus, they can significantly improve patient outcomes across various clinical settings [9,10].

The growing prevalence of chronic diseases such as diabetes, cardiovascular disorders, and respiratory conditions necessitates innovative solutions for effective patient diagnosis and treatment. Traditional methods, while reliable, often fail to deliver the rapid response needed to prevent complications or adjust therapies in real time. Intravascular biosensors, through their flexibility and high biocompatibility, offer much better integration with biological tissue [11]. This innovation is underpinned by advancements in micro- and nanotechnology, enabling the miniaturization and enhanced sensitivity of these devices [12].

Despite numerous reviews summarizing general advances in biosensor technologies, there is a notable lack of comprehensive analyses focusing specifically on intravascular biosensors and their integration with micro- and nanotechnologies. Existing reviews often address biosensors in broader contexts, without emphasizing the unique challenges and opportunities associated with implantable intravascular devices. This review aims to fill this gap by providing a critical assessment of emerging micro- and nanoscale innovations dedicated to intravascular applications, discussing not only technological advancements but also clinical translation barriers, biocompatibility concerns, and future trends such as biodegradable materials and AI-based data analysis. Our goal is to offer a structured and forward-looking perspective on how next-generation intravascular biosensors can revolutionize continuous health monitoring and personalized medicine.

**Figure 1:**
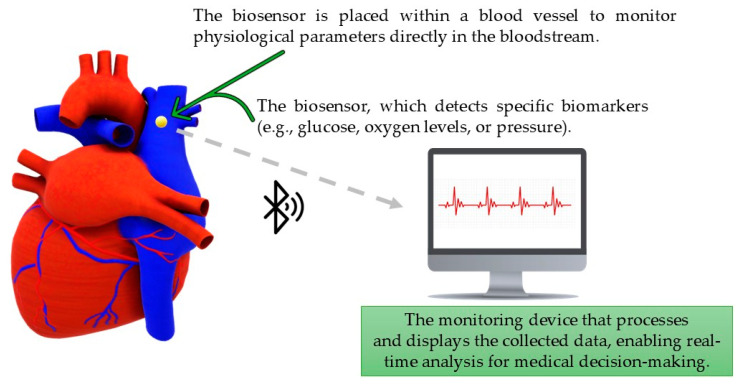
Diagram of intravascular biosensor functionality.

The diagram below (Figure 1) presents a schematic representation of the functionality of intravascular biosensors.

This solution provides the continuous and precise monitoring of physiological parameters, which is crucial for personalized medicine.

The scope of applications for intravascular biosensors is broad and includes glucose monitoring for diabetes management, the real-time tracking of blood oxygen levels, and the assessment of cardiovascular health through continuous blood pressure and pulse measurements [13,14]. Importantly, beyond diagnostics, these biosensors play a key role in therapeutic interventions, such as drug delivery and the personalization of treatment regimens based on the patient’s dynamic physiological responses [15]. This synergy between monitoring and treatment represents a paradigm shift towards truly personalized medicine.

An interesting solution that enables the monitoring and assessment of cardiorespiratory function is a photonic smart wristband developed by Li et al. [16]. An all-polymer sensing unit forms the basis of this wearable device, which constantly measures critical health metrics, such as blood pressure, heart rate, and respiration rate. Furthermore, the conducted research showed that the biometric identification process reached a correct rate of 98.55%, confirming its applicability in personalizing healthcare services.

The table below (Table 1) provides a detailed comparison of various types of biosensors, categorized by their applications, advantages, and disadvantages, alongside relevant references for each type.

**Figure 2:**
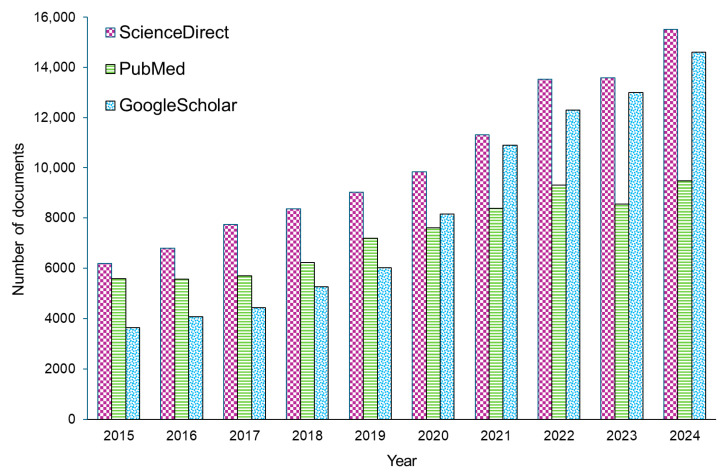
Number of documents collected from the ScienceDirect, PubMed, and Google Scholar databases from 2015 to 2024.

The number of published papers found in the ScienceDirect and Google Scholar databases after applying “biosensor” as a keyword has steadily increased over the last ten years, as shown in Figure 2. The graph shows different types of articles—both research and review articles, as well as other documents, including book chapters, short communications, and conference abstracts. It is worth mentioning that the number of relevant documents indexed in the database in 2025 was nearly 2.5 times higher compared to the number recorded in 2015. However, it should be noted that applying “intravascular biosensor” as a keyword yielded only 121 results in the database, dating back ten years. For instance, in 2021 and 2023, the number of documents meeting the criteria was 6 and 23, respectively.

Although researchers have presented many reviews focusing on various aspects of biosensors, there is no review relating to intravascular biosensors. Therefore, this paper provides a literature review on the current state of intravascular biosensors, with a focus on their technological foundations, clinical applications, and future potential. In more detail, following this introduction and general overview regarding biosensors (Section 1), specific areas of application, such as glucose monitoring, oxygen level assessment, and cardiovascular parameter monitoring, are described (Section 2). Progress achieved in disease diagnostics is then discussed, considering the detection of disease biomarkers and infection diagnostics (Section 3). Next, modern approaches to drug therapy monitoring and systems, as well as micro- and nanotechnology in intravascular biosensors, are presented, along with the role of emerging technologies like quantum dots and bioresorbable stents (Section 4 and Section 5). Furthermore, the possibility of applying biosensors for cancer diagnosis and treatment is discussed (Section 6). This work aims to highlight their transformative potential in modern medicine [25].

## 2. Monitoring of Physiological Parameters

Monitoring physiological parameters plays a crucial role in the diagnosis and treatment of various medical conditions. The analysis of biomarkers provides valuable insights into a patient’s health status, enabling timely intervention and, most importantly, personalized treatment. Advances in the field of biosensors have revolutionized healthcare through innovative solutions that combine precision and real-time monitoring. From detecting fluctuations in glucose levels in diabetic patients to assessing oxygen levels and monitoring cardiovascular parameters, biosensors address a wide range of clinical needs. These technologies not only enhance diagnostic accuracy but also improve therapeutic outcomes through integration into patient care workflows. However, their applications extend beyond acute cases, as they support long-term health monitoring and proactive disease prevention. As these systems continue to evolve, their scope of application is constantly expanding, promising a transformative impact on modern medicine. Additionally, this creates a significant research niche for scientists working on the development and optimization of these materials.

### 2.1. Glucose Level Monitoring

The blood glucose level is one of the most important indicators of a patient’s health status. The high prevalence of diabetes has led to extensive research into various glucose measurement methods to ensure continuous and accurate glucose monitoring. Although direct blood glucose monitoring provides precise information, many patients, particularly younger ones, are reluctant to undergo needle pricks for blood sampling due to the invasiveness of the procedure. An alternative is the use of an implanted glucose sensor. While this method is invasive, requires regular sensor replacement due to its limited lifespan, and carries the risk of microthrombus dissemination, it reduces the need for frequent and burdensome blood sampling for patients [26,27]. However, significant challenges, such as the long-term stability of sensors and calibration problems caused by the harsh environment inside the human body, must still be addressed, requiring the development of new and optimized solutions in the future [28].

Electrochemical implantable biosensors, such as subcutaneous or intravascular sensors, can provide real-time glucose level data, allowing for precise therapy adjustments [29]. David A. Gough et al. conducted a study monitoring glucose concentrations in subcutaneous tissue in diabetic patients using an implanted sensor/telemetry system. This sensor was based on membranes containing immobilized glucose oxidase and catalase, coupled with oxygen electrodes and a telemetry system integrated as an implant. Since no independent standard exists for sensor signals indicating dynamic glucose levels in tissues, a model was applied to describe the relationship between blood glucose levels and sensor signals. The tested sensor enabled long-term glucose monitoring, and the obtained results correlated with reference values, highlighting its potential to facilitate diabetes management [30]. Another study utilized an intravascular continuous glucose monitoring system in critically ill patients in intensive care units. This is particularly significant because both hyperglycemia and hypoglycemia are associated with adverse clinical outcomes in critical care patients. The study employed the GluCath System, which uses a chemical fluorescence quenching mechanism for optical blood glucose measurement via insertion into the radial artery or directly into a peripheral vein through a catheter. The GluCath System demonstrated acceptable accuracy during 48 h placement in the radial artery in post-cardiac surgery patients in intensive care units [31]. Another example of a continuous intravascular glucose monitoring system is the one developed by GlySure Ltd. This system offers continuous intravascular glucose monitoring using a diboronic acid-based receptor for precise plasma glucose measurement. Inserted via a central venous catheter, it measures glucose every 15 s. The sensor features an optical fiber within a sheath (<0.6 mm) and a chamber with glucose-detecting elements in hydrogel, protected by dialysis and microporous membranes. Advanced filtration minimizes interference from blood components, ensuring accuracy. The GlySure CIGM system has proven reliable, safe, and highly accurate in clinical settings [32]. Although progress in biosensor technology for diabetes management is encouraging, issues like precision, patient compliance, and affordability remain. Moving forward, efforts will focus on enhancing sensor precision, optimizing data analysis algorithms, and tackling challenges related to cost and accessibility [33]. While subcutaneous and intravascular sensors show promising accuracy, issues like biofouling, limited sensor lifespans, and signal drift remain unresolved, necessitating further work on antifouling coatings and sensor recalibration strategies.

A key technological parameter is the limit of detection (LOD) in intravascular blood and the linearity of the measurement. The standard physiological blood glucose concentration in a healthy person ranges from approximately 70 to 105 mg/dL [34]; therefore, sensors intended for in vivo measurement must be sensitive in the range of at least 1–10 mmol/L. Microneedle enzymatic biosensors have achieved an LOD of 1–7 µmol/L, corresponding to a concentration of 0.018–0.126 mg/dL. These sensors exhibit a linearity range of 0.05 to 9 mmol/L, which allows for the accurate mapping of glycemia changes in real time, including in situations of hypoglycemia and hyperglycemia [35].

Enzymatic and optical biosensors are most commonly used for glycemia. A more detailed discussion of biosensor technologies, including MEMS, is provided later in this section.

### 2.2. Oxygen Level Monitoring

Physiological processes such as circulation, respiration, digestion, and many others change the oxygen concentrations in the tissues and the blood [36]. Disturbances in the body’s homeostasis caused by reduced oxygen levels lead to respiratory and cardiovascular diseases. Hypoxia results in cellular dysfunction and organ failure, affecting critical organs such as the brain and heart. Monitoring oxygen levels in the body is a key element in patient diagnostics [37,38]. Oxygen saturation is an indicator that reflects the efficiency of oxygen transport to tissues and gas exchange [39]. The measurement is non-invasive and can be performed in both home and hospital settings. It is conducted using a pulse oximeter, which utilizes the phenomenon of spectrophotometry. The analysis is based on the absorption of red and infrared light, which penetrates tissue structures [40]. Pulse oximetry enables the diagnosis of conditions such as COVID-19 and hypoxia, which, despite low oxygen levels, may not present with symptoms like shortness of breath. Early detection helps to reduce the risk of complications [41]. The rapid and accurate monitoring of the local oxygen concentration in 3D tissue cultures allows the oxygen concentration to be controlled so that both healthy and pathological environments can be reproduced. For example, the 3D culture and oxygen monitoring system presented by Rivera et al. [36] consists of a simple design to remotely monitor oxygen concentrations during tissue culture. The researchers integrated a photonic oxygen sensor into a 3D tissue scaffold and regulated the oxygen concentration by controlling the flow of the purification gas. Although non-invasive oxygen monitoring technologies show significant promise, limitations such as calibration drift, motion artifacts, and low penetration depths in certain tissues still need to be addressed for broader clinical adoption.

Although optical photonic biosensors dominate in this case, the technologies used—as in the case of other parameters—will be discussed later in this section.

### 2.3. Intravascular Lactate Biosensors

Lactate is marker of hypoxia. Under non-steroidal conditions, it switches to anaerobic metabolism, which leads to the selective production of lactate by lactate dehydrogenase. Schierenbeck et al. [42] conducted a study on the implementation of intravascular microdialysis for the continuous monitoring of lactate levels in patients undergoing cardiac surgery. The continuous monitoring of blood lactate levels was performed in 80 patients undergoing cardiac surgery using microdialyzers placed in the superior vena cava. Single- or triple-lumen central catheters with microdialysis capabilities were used for this purpose. Arterial blood samples were simultaneously collected hourly for blood gas analysis, providing reference values for comparison. A total of 1601 pairs of lactate samples were collected, analyzed by microdialysis and traditional blood gas analysis. The results confirmed that central venous microdialysis is a precise and reliable method for the continuous monitoring of lactate levels in cardiac surgery patients. This technique may also be useful for the early detection of perfusion disorders in critically ill patients. Yin Ho et al. [43] developed a 5 Fr intravascular nitric oxide (NO) catheter containing a sensor for continuous lactate measurement and a port for a pressure sensor. The device exhibits antibacterial and antithrombotic properties and can be implanted intravenously. In an in vivo study of six piglets undergoing cardiac surgery with cardiopulmonary bypass (CPB), sensors were placed in the femoral vessels and in the CPB circuit. Lactate measurements obtained from the sensors in the CPB were consistent with blood gas measurements, whereas the sensors in the femoral arteries correlated well with measurements only before CPB. The pressure sensors provided accurate readings, comparable to those of FDA-approved devices. The authors recommend implanting sensors in the CPB circuit for the monitoring of lactate levels and in peripheral arteries or veins before and after CPB. This study supports the usefulness of this system for continuous metabolic monitoring during infant cardiac surgery.

### 2.4. Blood Pressure and Heart Rate Monitoring

Blood pressure and pulse monitoring is crucial for cardiovascular health, as it plays a vital role in diagnosing and preventing diseases that can be fatal for patients. Hypertension, often referred to as the “silent killer”, and arrhythmias remain among the leading causes of morbidity and mortality worldwide [44]. For this reason, the scientific community continues to seek solutions to enhance the fight against these conditions. Intravascular biosensors have emerged as a groundbreaking solution, offering precise and continuous monitoring capabilities that surpass those of traditional methods [45]. In recent years, intravascular blood pressure and heart rate sensors have leveraged miniaturized MEMS and piezoelectric technologies to achieve continuous real-time monitoring. For instance, sensors fabricated on a polymer substrate must be in contact with the catheter surface to achieve a better signal and greater durability. The authors of [46] describe a study in which gold patterns were fabricated on a polyimide surface, and a micrometer-sized SU-8 pressure chamber was mounted to transduce pressure. Additional gold patterns were fabricated as a resistive temperature detector, and a tensimeter was used to measure pressure. An in vivo experiment in a mouse model demonstrated that the catheter-mounted sensor could measure heart rate and carotid blood pressure in real time. A subsequent publication [47] discussed various sensors for the monitoring of cardiovascular parameters, including heart rate. These devices enable the detection of pulse waves generated by blood vessels with very high sensitivity (below 10 kPa), making them suitable for wearable applications. Triboelectric nanogenerators function as self-powered biosensors—they convert body movement into an electrical signal, enabling continuous heart rate monitoring without the need for external power. While this section outlines the clinical relevance of these parameters, the following subsection focuses in more detail on technological platforms enabling advanced hemodynamic monitoring.

### 2.5. Technological Platforms for Advanced Biosensing

Recent advances have led to the development of a new generation of biosensors capable of precisely monitoring physiological parameters in real time. Among the most influential technologies are MEMS, nanomaterials, quantum dots, and bioresorbable platforms, each bringing unique functionalities that enhance diagnostic accuracy and therapeutic efficacy.

Microelectromechanical systems (MEMS) represent a class of miniaturized devices that integrate mechanical and electronic components. Their unique properties, such as high sensitivity, miniaturization, and real-time signal processing, make them a versatile tool in biosensing platforms, including cardiovascular monitoring. Modern intravascular biosensors utilize advanced microelectromechanical systems (MEMS) and nanotechnology to achieve exceptional sensitivity and precision. These devices combine pressure-sensitive membranes with piezoelectric or capacitive sensing elements, which have the ability to detect subtle changes in blood vessel walls corresponding to the pulsatile blood flow [48]. Advances in wireless technology further enable real-time data transmission, facilitating seamless integration with patient monitoring systems [49].

**Figure 3:**
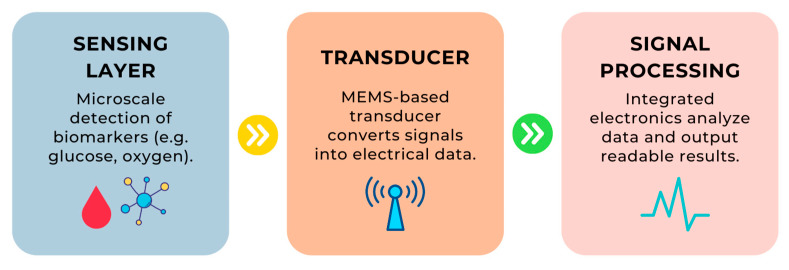
A diagram illustrating micro-electromechanical systems (MEMS) technology used in biosensors, highlighting its three main operational stages.

The following table (Table 2) presents a summary of various technologies used in biosensors, highlighting their applications, advantages, examples, and relevant references. It illustrates the versatility of technologies such as MEMS and nanomaterials in advancing biosensor functionalities for diverse medical and diagnostic applications. Additionally, the operation of MEMS technology in biological sensors is illustrated in Figure 3.

Biosensors integrated with bioresorbable stents can offer dual functionality: firstly, they ensure arterial patency; secondly, they enable the localized measurement of hemodynamic parameters. Such biosensors are coated with biocompatible materials, which minimize inflammatory responses, enhance patient safety, and improve device durability [58]. Such technologies highlight the shift towards multifunctional devices that align with the principles of precision medicine. Future intravascular devices will likely combine multiple functionalities, including sensing, drug delivery, and real-time AI-driven data interpretation, paving the way for fully autonomous diagnostic–therapeutic platforms.

How does it work in practice?

Step 1: A biomarker (e.g., a glucose molecule) interacts with the sensing layer, which recognizes its presence.
Step 2: Information about this interaction is converted into a signal by the MEMS transducer.
Step 3: The signal is processed by the signal processor, which ultimately delivers the result in an understandable format.

MEMS technology enables the miniaturization of these devices, making them suitable for high-precision medical applications, such as intravascular biosensors or medical implants. Studies have shown that the use of these biosensors enables the earlier detection of hemodynamic abnormalities, which translates into faster intervention and improved treatment outcomes [59,60].

In perioperative care, where maintaining cardiovascular stability is critical, intravascular biosensors provide real-time data, enabling the precise management of anesthesia and fluid therapy. In high-risk surgeries, such as cardiac procedures, the use of these biosensors allows for the highly accurate monitoring of hemodynamic parameters, minimizing the risk of complications [61].

Furthermore, the integration of intravascular biosensors with wearable or implantable devices supports long-term patient monitoring, which is crucial for the early detection of conditions such as heart failure or vascular stenosis. Studies indicate that the continuous monitoring of hemodynamic parameters using these biosensors enables the earlier detection of deteriorating cardiac function, facilitating the quicker implementation of appropriate therapeutic interventions [62]. Despite technological advancements, the long-term stability, miniaturization, and reliable wireless data transmission of intravascular pressure biosensors remain critical technical hurdles that need focused research efforts.

Despite their promising potential, several challenges still need to be addressed to advance diagnostics to the next level. Issues such as device miniaturization, long-term biocompatibility, and power supply limitations remain significant barriers [63]. Research into self-sustaining power mechanisms, such as harvesting energy from blood flow, represents a promising path to overcoming these obstacles [64].

Additionally, scientific reports have considered the integration of artificial intelligence and machine learning algorithms into biosensor platforms [65]. The possibility of integrating artificial intelligence algorithms with systems applied to the continuous monitoring of glucose constitutes an extremely important topic, which could change the futures of many patients with chronic diseases [66]. The analysis of huge amounts of data and identifying existing correlations and patterns afterwards can be accomplished using AI technology, whereas these tasks are usually too demanding or time-intensive for manual processing. Similarly, processing noisy and multimodal signals obtained by biosensors can be facilitated with machine learning models, which in turn improve the detection of pathological conditions [67]. Omar et al. presented an Al-based prediction model, which showed that the fusion of information coming from multifunctional sensors is possible [68]. Jin et al., in their work [69], concluded that there are obstacles that prevent biosensors from becoming popular, such as inaccuracy and consumable costs.

Moreover, the application of other biodegradable materials could offer a solution for the development of transient biosensors that degrade after fulfilling their diagnostic or therapeutic roles. The selection of appropriate materials is a key aspect. For instance, materials such as PLGA or silk fibroin are biodegradable. However, they are prone to swelling simultaneously, which hinders their application in biosensors. On the other hand, polylactic acid (PLA) or biodegradable magnesium alloys can ensure the functionality of devices within an intended lifespan, as well as being safe for the human body. This approach would eliminate the need for an additional step involving device removal [14,68,70,71].

### 2.6. Advantages of Intravascular Biosensors Compared to Conventional Biosensing Platforms

Intravascular biosensors, by virtue of their placement directly within blood vessels, offer several key advantages over conventional biosensing platforms, such as subcutaneous, surface-mounted, or wearable systems. Their strategic location enables the following:

- Direct access to circulating biomarkers, allowing for more accurate and real-time measurements [32,72];
- Faster response times, which are crucial in dynamic clinical settings such as intensive care or surgery [12];
- Higher clinical relevance of measurements, particularly for drugs or metabolites that exhibit compartmentalization (e.g., plasma vs. interstitial fluid) [73];
- Integration with delivery systems (e.g., infusion pumps, stents), enabling closed-loop therapies [49].

Despite these advantages, intravascular sensors face challenges related to biocompatibility, the risk of thrombosis, sensor drift, and power and communication limitations [74,75]. Table 3 below summarizes these features.

The outlined comparison sets the stage for examining specific implementations of intravascular biosensors that demonstrate their clinical relevance and technological maturity.

## 3. Disease Diagnostics

Disease diagnostics enables the identification and monitoring of inflammatory states and changes within the body. Access to advanced technologies enhances and expands the capabilities of diagnostic methods. Techniques such as spectroscopy, fluorescence, and DNA analysis are utilized to identify biomarkers of various conditions and differentiate between them. The fluorescent response plays a crucial role in clinical in vivo medical analysis, allowing the precise monitoring of biological processes. Two key areas of diagnostics are distinguished: the detection of biomarkers and the identification of infections caused by pathogenic agents [82,83,84].

### 3.1. Detection of Disease Biomarkers

Biomarkers are indicators that reflect the health status of an organism. They are used in detecting, monitoring, and evaluating responses to therapy, providing information about physiological processes occurring in the body. Biomarkers include metabolites, proteins, DNA, and RNA. They are detected in tissues and bodily fluids, primarily blood. The identification of biomarkers has been made possible through advancements in methods such as PCR, mass spectrometry, and genomic analysis, enabling rapid diagnostics in cardiovascular diseases and cancer [85,86]. Multimarker test technologies like multiplex PCR and next-generation sequencing (NGS) allow for therapy personalization by simultaneously detecting multiple biomarkers [87]. Table 4 summarizes key biomarkers and their diagnostic applications, detection methods, and associated advantages, highlighting their significance in modern healthcare. In cancer diagnostics, biomarkers are used to monitor tumor progression through next-generation sequencing, which detects genetic mutations and tumor-specific gene expression. In Alzheimer’s disease, protein analysis can enable diagnosis at the early stages of nervous system pathology. A challenge in diagnostics is that a single biomarker may be associated with multiple conditions, complicating the identification of specific diseases [88,89,90]. For example, Zhong et al. [91] have developed a fast and highly sensitive aptasensor for the quantification of biomarkers of amyloid-β (Aβ) oligomers, which are identified as reliable biomarkers for the diagnosis of Alzheimer’s disease (AD). With the advantages of low consumption, simple operation, limited time requirements, and high sensitivity, this new aptasensor driven by hyperbranched rolling circle amplification (HRCA) has great potential in the early diagnosis of AD.

The data presented in Table 4 showcase the versatility of biomarkers in diagnosing and managing various medical conditions. Glucose monitoring plays a critical role in diabetes care, leveraging electrochemical biosensors for fast and accurate detection. Troponin serves as a vital indicator for myocardial damage, offering high specificity through immunosensors. CRP facilitates the rapid detection of inflammation and infections using biochemical tests and biosensors. Additionally, genetic mutations analyzed through NGS and PCR enable early cancer diagnostics and therapy personalization. Collectively, these biomarkers underscore the transformative potential of advanced detection technologies in improving diagnostic accuracy and patient outcomes. Biosensors using surface plasmon resonance (SPR) technology are also used for CRP applications. TN detection using SPR is one of the most interesting applications in the diagnosis of acute myocardial infarction. Typically, SPR-based TN detection tools can be classified into two types: immunosensors and aptasensors. Nanostructures have an improved LOD. Furthermore, SPR-based biosensors can be fabricated using nanostructures. To date, SPR-based TN biosensors have been developed as immunosensors [101]. In modern biosensors used to measure troponin, based on field-effect transistors (FETs), a semiconductor channel material is connected between two metal electrodes, and the channel material is functionalized with receptors. The analysis time for FET-based biosensors is significantly reduced thanks to direct electrical detection without additional labeling. This simple and rapid diagnostic method is ideal for detecting a cardiac marker of myocardial infarction, where early diagnosis and rapid treatment are required. Electrochemical FET-type immunosensors based on silicon nanofibers (SiNW-FETs) can detect cTnI with an LOD of only ~5 pg/mL in buffer and 10 pg/mL in whole blood [102].

### 3.2. Infection Diagnostics

Human achievements in the medical field are considered to be significant. Nevertheless, currently, infectious and contagious diseases still pose a serious threat to human life. It is estimated that about 15% of deaths registered worldwide are caused by communicable diseases [103]. Some viral diseases, such as Human Papillomavirus (HPV), HIV, and SARS-CoV-2, can spread rapidly around the world, resulting in a variety of symptoms in affected individuals [104].

**Figure 4:**
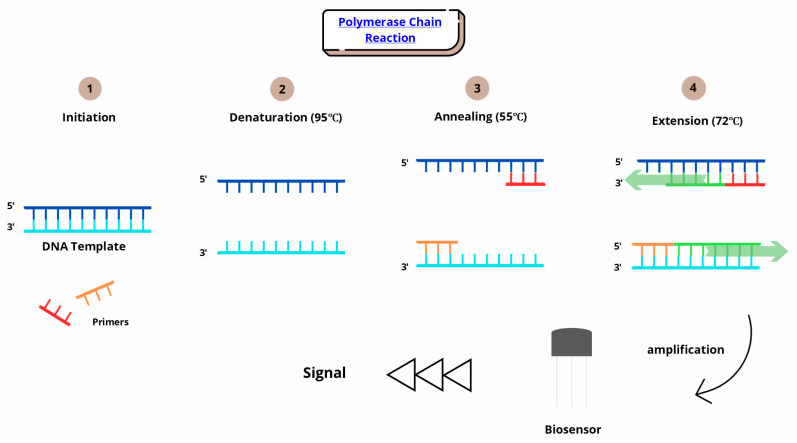
Schematic of the biosensor principle.

Infection diagnostics involves identifying pathogens that can lead to infections and exacerbate the occurrence of chronic diseases. Traditional diagnostic methods are time-consuming and rely on microbiological techniques, such as culturing fungi, viruses, or bacteria on selected media. Modern methods are more precise and, above all, faster. Pathogen DNA detection is enabled through rapid, highly sensitive PCR and RT-PCR tests. Multiplex PCR is also used, allowing the simultaneous detection of multiple pathogens [105,106]. Biomarkers play a crucial role in infection diagnostics, and their rapid detection is particularly important in cases of sepsis and rapidly progressing infections [107]. The PCR method involves a series of chemical reactions that lead to the copying of specific nucleic acid sequences. PCR makes it possible to identify the genetic material of a pathogen. When the sample is introduced into the biosensor and then the bioreceptor interacts with the target molecule, a transducer in the biosensor converts the changes into a signal that can be used to quantify the elements in the sample (Figure 4).

Kumar et al. [108], in their work, successfully developed and optimized an assay designed to detect anti-SARS-CoV-2 N antibodies, indicating prior SARS-CoV-2 infection. It is a visual test that uses gold nanoparticles (GNPs) functionalized with a peptide dendrimer. This idea enables direct detection with high sensitivity for breakthrough infections, not only in humans but also in animals.

## 4. Modern Approaches to Drug Therapy Monitoring and Systems

The world faces a significant challenge in providing healthcare and combating global threats such as Alzheimer’s disease, cancer, cardiovascular diseases, mental disorders, stroke, AIDS, and COVID-19. In addition to user-friendly diagnostics, there is an urgent need for treatment methods that are more patient-friendly. Traditional surgical treatments carry risks, require prolonged recovery periods, and incur high costs. In this context, implantable devices offer an efficient and convenient alternative [109].

### 4.1. Drug Therapy Monitoring

Therapeutic drug monitoring (TDM) is a clinical practice involving the measurement of pharmaceutical drug concentrations in patients’ biofluids at designated intervals to enable accurate and timely dose adjustments [110,111]. There are many drugs that have narrow and even different therapeutic windows for various cases, and one of them is methotrexate. It is used to treat patients with autoimmune diseases, such as rheumatoid arthritis (RA) [112,113].

Another instance of a drug that is characterized by a narrow therapeutic window is imatinib; this is a tyrosine kinase inhibitor that is used in the treatment of chronic myeloid leukemia. Researchers have indicated that excessive levels of imatinib increase the risk of adverse effects, whereas subtherapeutic levels may cause relapse or the development of resistance. Therefore, its clinical efficacy depends heavily on keeping the drug concentration at the proper level [114,115,116].

Biosensors represent a class of analytical tools capable of the rapid and precise determination of therapeutic drug concentrations. Significant advancements in instrumental platforms and detection methods, and the development of nanobiosensors, have greatly expanded the potential applications of these devices in drug therapy monitoring [117]. Drugs with a narrow therapeutic index often present a significant challenge because their therapeutic doses frequently overlap with toxic levels, increasing the risk of adverse effects. As a result, their administration requires meticulous attention, involving constant monitoring and the detailed analysis of their pharmacokinetic and pharmacodynamic properties [118].

Applications of biosensors in the monitoring and delivery of anticancer drugs are discussed in detail in Section 6.

In Table 5, examples of drugs administered using intravascular biosensors are summarized, along with information about the disease or condition treated, the type, and the most important features of the applied biosensor.

The table above demonstrates the versatility of intravascular biosensors in drug delivery and monitoring across various medical applications. These biosensors enable the real-time and precise control of therapeutic agents, improving treatment efficacy and minimizing adverse effects. Optical biosensors enhance immunosuppressant management in transplant medicine. These technologies demonstrate significant potential in advancing personalized medicine through precise and responsive drug delivery systems.

### 4.2. Drug Delivery Systems

Biosensors combined with drug delivery systems (DDS) are widely used in various diseases and health conditions, such as diabetes, Parkinson’s disease, respiratory diseases, or cardiovascular diseases, due to their ability to deliver drugs locally, the possibility for personalized therapy, fast action, and the administration of the proper dosages of drugs. This modern approach, offering combined systems, necessitates the development of a device that has a closed-loop system. For instance, in the management of diabetes, biosensors continuously monitor blood sugar levels and, on this basis, ensure the appropriate dose of insulin, thereby reducing the probability of both hyperglycemia and hypoglycemia. Similarly, one of the key challenges in Parkinson’s disease patients is to provide an appropriate supply of dopaminergic drugs, such as levodopa, to optimize symptom management [124,125,126,127].

**Figure 5:**
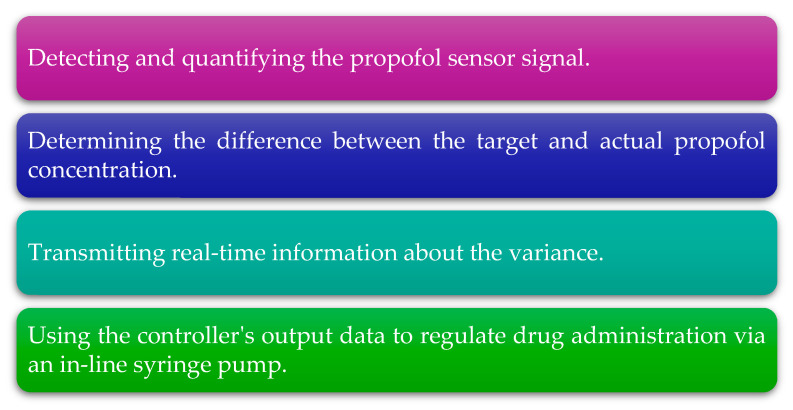
A diagram illustrating the information obtained for the prototype of a “smart” intravenous catheter with an integrated biosensor.

Implantable systems that enable on-demand or self-regulated drug delivery are now feasible by utilizing internal and external stimuli for drug administration to the ocular, gastrointestinal, and subcutaneous regions [128]. One potential application of biosensors is their use in the dynamic delivery of propofol for total intravenous anesthesia. A prototype of an “intelligent” intravenous catheter equipped with a biosensor has been developed, capable of quantitatively determining the amount of propofol in the blood in real time. The proposed method and the biosensor used in the study allow for rapid and stable drug detection, and it delivers numerous valuable insights, as described in Figure 5 [81].

Bruchas et al. described wireless optofluidic probes, outlined in their article as a promising technology for precise drug delivery and the manipulation of deep brain tissue in freely moving animals. These probes combine the functions of advanced in vivo pharmacology and optogenetics into a single, soft implant. They address the limitations of traditional devices, such as metal cannulas and fiber optics, by enabling simultaneous drug and light delivery to specific areas of the brain, allowing the targeting of the same cells with both drugs and photostimulation [129].

### 4.3. Examples of Intravascular Sensor Implementations

The clinical relevance of intravascular biosensors is closely tied to their ability to directly access circulating biomarkers and provide real-time measurements under dynamic physiological conditions. In recent years, several systems have been developed or tested to demonstrate the viability of biosensor integration directly into the vascular system, particularly for critical care and perioperative monitoring.

One of the most prominent examples is the GluCath intravascular continuous glucose monitoring system developed by GlySure Ltd., which utilizes a fluorescence quenching-based sensing mechanism embedded in an optical fiber. The system is designed to be introduced into a central venous catheter and enables continuous glucose monitoring at the plasma level. Clinical studies have validated its performance in intensive care unit (ICU) settings, particularly in patients undergoing cardiac surgery, where strict glucose control is crucial in reducing postoperative complications [77,130]. However, the requirement for central venous catheterization limits its use to inpatient settings such as the ICU, restricting its broader applicability in outpatient or ambulatory care environments.

Beyond glycemic control, lactate monitoring via central venous microdialysis represents a critical advancement in metabolic surveillance. In a landmark study, Schierenbeck et al. implanted microdialysis catheters into the superior vena cava during cardiac surgery, enabling continuous lactate measurements. This approach provided the early detection of tissue hypoperfusion before systemic signs appeared, proving invaluable in perioperative risk management [42]. This technique, while valuable, is invasive and technically complex, which may hinder its widespread use outside of specialized surgical settings.

A further innovation involves dual-analyte electrochemical microcatheters for the simultaneous monitoring of propofol and fentanyl, as demonstrated by Moonla et al. This platform embeds two chemically distinct carbon-paste working electrodes within a narrow Teflon tube: one optimized for propofol detection in the μM range and the other for fentanyl in the nM range. Reference electrodes, antifouling coatings, and voltammetric techniques enable continuous real-time detection in artificial plasma and whole blood matrices, highlighting their feasibility for closed-loop anesthesia delivery [123]. Despite its promising sensitivity, the platform remains in the preclinical stage and faces challenges related to long-term stability, miniaturization, and clinical validation.

In addition, recent advances in transient electronics have opened the door to truly biodegradable intravascular sensors. In a foundational review, Fanelli and Ghezzi describe platforms built from biodegradable materials and designed to operate wirelessly for a limited duration before safely degrading in vivo. These systems support the transient monitoring of physiological parameters such as pressure or biochemical markers, eliminating the need for surgical retrieval and mitigating long-term implant safety concerns [131]. For example, the authors describe a fully degradable pressure sensor fabricated from magnesium and polylactic-co-glycolic acid (PLGA), capable of operating wirelessly in vivo for over two weeks before bioresorption. This system exemplifies the potential of transient electronics in vascular applications, although it remains limited by fabrication complexity and power delivery constraints.

These representative implementations clearly demonstrate the practical viability and clinical potential of intravascular biosensors. From glucose and lactate monitoring to anesthetic drug detection and the emergence of biodegradable electronic systems, recent advances underline the versatility of biosensor technologies within the vascular environment. Their ability to provide continuous, real-time, and physiologically relevant data directly from the bloodstream positions intravascular biosensors as a transformative component in the future of critical care, perioperative monitoring, and personalized medicine.

## 5. Micro- and Nanotechnology in Intravascular Biosensors

The application of micro- and nanotechnology enables the development of sensors with very small dimensions. An additional advantage is the possibility of directly introducing biosensors into blood vessels, which reduces the risk of complications. Nanofibers are used in electrochemical sensors to detect cardiac biomarkers, providing information about myocardial damage, while microsensors can monitor blood components [132]. Surfaces coated with nanomaterials can respond specifically to substances such as glucose or cholesterol. Carbon nanotubes and graphene, characterized by a large active surface area and excellent conductivity, are used to detect very low analyte concentrations. Micro- and nanosensors monitor critical blood parameters, such as glucose levels, oxygen concentration, and inflammatory markers. These are particularly important in diagnosing diabetes and cardiovascular diseases. Electrochemical biosensors utilize nanostructures to enhance the detection sensitivity. Upon interaction with specific biomarkers, quantum dots based on nanotechnology emit light [133,134]. Biocompatible coatings consisting of nanomaterials are hydrophobic and prevent thrombosis on sensors and protein deposition. Additionally, biosensors can deliver drugs, thus responding to changes in a patient’s health condition [135,136].

### 5.1. Stents and Medical Implants

Stents are a group of medical devices designed to maintain the patency of blood vessels. They are most commonly produced from polymers or metals and are used in cardiology to treat cardiovascular diseases by preventing vascular narrowing. Modern stents are additionally coated with nanoparticles to enhance their durability [137,138]. A revolutionary development is the introduction of biodegradable stents, which decompose after vessel regeneration. However, the adoption of advanced stents and the use of nanoparticles face delays in market introduction due to the lack of specific regulatory guidelines. This is a complex and layered task owing to the necessity of overcoming multiple clinical translation barriers. Existing regulations aim to ensure the safety, reliability, and effectiveness of implemented solutions. Therefore, there is an obligation to demonstrate that the applied materials do not cause toxicity, inflammatory conditions, or thrombosis during their intravascular application. Modern implants enable the monitoring of biological parameters and the early prevention of pathological developments through drug release. Furthermore, coating structures with silver or copper nanoparticles reduces the risk of infections. These coatings can also support tissue regeneration by releasing appropriate substances or inhibiting their degradation [139,140]. For example, Kim et al. [141] presented an implantable polymer-based temperature sensor for dental applications in their work. It has the advantage of being able to transmit real-time warning signals, making it possible to detect a problem before the sensor even fails. The fabricated sensor showed high linearity, repeatability, and stability under high-stress conditions caused by dynamic temperature changes. This solution ensures appropriately matched diagnostics to the detected ailment.

### 5.2. Nanoparticles in Imaging

Nanomaterials play a significant role in improving the sensitivity and stability of biosensors, as they provide a large active surface area for the immobilization of biomolecules. This results in the higher performance of biosensors [142]. Cui et al., in their work [143], developed a metallene structure for a gold-based electrochemiluminescence (ECL) biosensor for the detection of coronary artery calcification (CAC). Luminescent nanobubbles were synthesized from copper nanoclusters (Cu NCs). The results showed that the developed biosensor has huge potential as an auxiliary biomarker for the diagnosis of coronary artery calcification diseases. Chen et al. [144] designed and fabricated a three-dimensional gold/ferrocene/liposome nanoparticle cluster (GFLC) as a component of an electrochemical biosensor. Gold nanoparticles are the enhancement component. Liposomes are the particle recognition component, and ferrocene is the signal input component. The GFLC module has been successfully applied to the electrochemical analysis of lipopolysaccharide (LPS). This is an excellent application example that can contribute to the development of biological sensors.

Nanoparticles have found extensive application in medical imaging diagnostics. Their size, typically below 100 nm, enhances the optical and magnetic properties. Magnetic resonance imaging (MRI) utilizes superparamagnetic iron oxide nanoparticles (SPIONs) to improve the image contrast. This is particularly important in imaging soft tissues, which have homogeneous properties compared to hard tissues. Soft tissues, such as organs, muscles, and fat, contain a high and similar amount of water, resulting in minimal contrast differences during imaging. Iron nanoparticles can bind to specific cancer markers, and they are biodegradable, being converted into safe forms during metabolism. Gold nanoparticles (AuNPs) are used in highly sensitive biosensors and imaging, similarly to quantum dots. They enable the detection of specific pathogens and molecular interactions [145,146]. Sharma et al. [147], in their work, described the synthesis of zinc sulfide nanoparticles coated with benzyldihydrazone-N,N′-bis(2-hydroxy-4-diethylamino-1-formylbenzene)m(BDH-DEHB@ZnS-NP) using the co-precipitation method. At certain doses, BDH-DEHB@ZnS-NP can be used as an anticancer drug, as well as a bioimaging sensor for Hg^2+^ ions, without inducing cytotoxic effects.

While intravascular biosensors present numerous clinical advantages, several critical technological hurdles remain:

- Miniaturization to avoid vascular occlusion and enable deployment via standard catheter systems. Recent reviews emphasize that implantable sensors must be dramatically miniaturized down to sub-millimeter form factors to avoid disrupting the blood flow or damaging vessel walls [148].
- Long-term biocompatibility, requiring advanced antifouling coatings (e.g., PEG, zwitterionic hydrogels). Chronic implantation often results in biofouling and immune encapsulation, degrading sensor performance. Antifouling surfaces such as zwitterionic polymer brushes have been shown to reduce protein adsorption by over 99% and preserve sensitivity in serum for >15 days [149].
- Reliable wireless data transmission from within deep vasculature to external receivers. Deep-tissue telemetry faces challenges related to signal attenuation and power constraints. Reviews note that implantable antennas and optical or RF-based wireless links require careful architectural design to ensure reliability [150].
- Energy autonomy, such as harvesting energy from blood flow or inductive coupling. Techniques like inductive coupling or ultrasound-based wireless power transfer are highlighted as feasible but limited by tissue depth and alignment requirements [151].
- Regulatory hurdles, especially for devices placed in high-risk cardiovascular sites. Implantable devices must meet stringent biocompatibility, sterilization, and safety standards, creating barriers for commercialization [80].

Recent approaches leveraging AI-assisted signal processing and biodegradable platforms show promise in extending the lifetimes and clinical reliability of intravascular devices. The comprehensive integration of sensing, data analytics, and actuation (e.g., drug delivery) will likely define the next generation of precision biosensing platforms.

## 6. Cancer Diagnosis and Treatment

In the current oncological sciences, it is important not only to detect cancer early but also to monitor therapy, especially for anticancer drugs acting in the therapeutic range. The real-time monitoring of in vivo drug levels enables feedback systems to regulate delivery rates, optimizing dosages to maximize efficacy and minimize toxicity. This closed-loop approach is particularly suited for prolonged intravenous treatments, such as those required in cancer therapy, although it remains underexplored in current research [125]. It has been demonstrated that combining diagnostic in vivo biosensors with drug delivery systems (DDS) for therapeutic agents allows the creation of theranostic platforms, leading to groundbreaking advances in the early detection and treatment of cancer [152]. Early detection and reliable diagnostics are key to the successful design of cancer therapies with better prognosis [153].

### 6.1. Biosensor Technologies and Their Diagnostic Applications

A promising diagnostic tool is represented by surface plasmon resonance-based biosensors, as they do not require the labeling or separation of cells. Cady et al. developed a lab-on-a-chip microarray biosensor using surface plasmon resonance coupled with grating and surface plasmon fluorescence to identify circulating tumor cells in a mouse model. While the focus was on blood analysis to detect circulating tumor cells, the method also allowed the analysis of many other components, such as cytokines, leukocytes, plasma antibodies, and heat shock proteins, on the same microarray [154]. This approach highlights the potential of SPR-based systems in minimally invasive cancer diagnostics.

Another interesting method was introduced by Jiang’s team, who developed an electrochemical TUNEL method using a three-dimensional biointerface for cytosensors, significantly enhancing the cell capture efficiency. By employing a quantum dot (QD)-based nanoprobe and electrochemical analysis, the sensor demonstrated high effectiveness in detecting apoptotic cells, positioning it as a promising tool for early cancer diagnostics and treatment monitoring [155].

### 6.2. Application of Nanotechnology in Cancer Diagnosis and Monitoring

The development of nanotechnology has led to the invention of a nanobiosensor capable of rapidly and sensitively detecting methotrexate at clinically relevant concentrations, offering potential for effective chemotherapy monitoring. Although the nanobiosensor was originally developed for drug monitoring, this approach demonstrates the sensitivity required for real-time biomarker tracking and theranostic applications in cancer care [156].

Quantum dots (QDs) are nanoscale semiconductors with tunable optical and electronic properties, making them excellent candidates for biosensing [157]. QDs conjugated with antibodies, aptamers, oligonucleotides, or peptides can target cancer markers. Their fluorescence allows QDs to serve as labels for in vitro biomarker assays and as potential in vivo imaging agents [158].

Graphene quantum dots (GQDs), a subclass of QDs, offer enhanced biocompatibility, solubility, and chemical stability. GQDs, due to their exceptional properties, are ideal for early cancer diagnosis when used with biomolecules that selectively recognize cancer biomarkers and convert them into detectable signals using optical, electrochemical, and chemiluminescent biosensors. These sensors enable the sensitive detection of key cancer biomarkers, including antigens, enzymes, hormones, and pH changes. GQD-based biosensors offer effective cancer diagnosis and enable the evaluation of anticancer therapy efficacy [159].

The application of advanced nanomotors equipped with biosensing capabilities and the ability to deliver drugs directly at the cellular level has the potential to transform chemotherapy in the near future. However, such applications remain largely unexplored and fall beyond the current diagnostic capabilities [125].

## 7. Conclusions

This study highlights the wide-ranging clinical applications of intravascular biosensors. Among them, advantages such as continuous glucose monitoring, oxygen saturation assessment, and cardiovascular parameter tracking can be distinguished, which significantly support the individualization of therapy in precision medicine. New developments in cancer diagnostics, such as SPR biosensors for the detection of circulating tumor cells and quantum dot cytosensors for the identification of apoptotic cells, demonstrate how intravascular biosensors are evolving to detect complex diseases. The conducted review of the world literature suggests that progress in the field of intravascular biosensors is leading to a revolution in healthcare. Technologies such as lab-on-a-chip, nanomaterial sensors (e.g., carbon and graphene quantum dots), and SPR systems are driving additional applications, particularly in oncology. Theranostic biosensors are a further development, followed by monitoring and treatment. This is possible through innovative solutions combining precision and the ability to monitor changes in real time.

Despite the immense potential of biosensor technologies, challenges remain, including long-term biocompatibility, miniaturization, and integration with existing medical systems. Key challenges remain the stability of sensor performance in vivo and protection against immunological degradation. Promising research directions include biodegradable coatings, energy-autonomous systems, and biosensors capable of wireless or integrated communication with infusion pumps. Future research should focus on overcoming these limitations, particularly in areas like autonomous powering and the integration of artificial intelligence algorithms for real-time data analysis.

In conclusion, intravascular biosensors offer remarkable opportunities for improvements in therapeutic outcomes, transforming the landscape of contemporary diagnostics and therapy through the close integration of real-time measurements and treatment. Their potential is particularly visible in personalized oncology, where technologies such as SPR-based cancer cell detectors, electrochemical cytosensors, or diagnostic platforms with GQDs can significantly improve early detection, therapy monitoring, and individualization. Their development will play a pivotal role in advancing personalized and precision medicine. Targeted multidisciplinary efforts combining materials science, bioengineering, and machine learning are urgently needed to overcome the current barriers and fully realize the potential of intravascular biosensors in clinical practice.

The examples and comparative analyses provided throughout this manuscript underscore the unique potential of intravascular biosensors to deliver high-precision, real-time data directly from within the circulatory system. This focused perspective reaffirms the relevance of micro- and nano-enhanced biosensing strategies specifically tailored to intravascular applications.

## Figures and Tables

**Table 1:** Comparison of different types of biosensors.

Type of Biosensor	Applications	Advantages	Disadvantages	Refs.
Electrochemical	Glucose and blood pressure monitoring	High sensitivity, broad applicability	Sensitivity to chemical interferences	[10,17]
Optical	Oxygen saturation measurement, biomarker detection	Safety, non-invasiveness	Limited long-term durability	[18,19]
Magnetic	Pathogen detection, cancer biomarker, immunoassays	High specificity, no optical background interference	Requires external magnet setups, limited commercial use	[20,21]
Acoustic (SAW, QCM)	Virus identification, small molecule and toxin sensing	Label-free, real-time, high sensitivity	Sensitive to environmental conditions and mechanical vibrations	[22,23]
Thermal	Enzyme activity, small molecule sensing	Simple readout, label-free	Low sensitivity, affected by ambient temperature	[24]

**Table 2:** Technologies used in biosensors.

Technology	Applications	Advantages	Examples	Refs.
**Microelectromechanical Systems (MEMS)**	Monitoring pressure, glucose, heart rate	Miniaturization, high sensitivity	Real-time monitoring in implants	[50]
**Nanomaterials**	Biocompatible coatings, biomarker detection, surface modification	Reduced thrombosis, precision, biocompatibility	Nanoparticles in stents and biosensors	[51]
Graphene and Carbon Nanotubes	Detection of low analyte concentrations	High surface area, conductivity	Electrochemical sensors in diagnostics	[52,53]
Quantum Dots	Fluorescence, cancer diagnostics	High sensitivity, multifunctionality	Imaging diagnostics and biomarker sensors	[54,55]
Metal Oxide Nanostructures	Enzyme sensors, electrochemical detection	Catalytic activity, chemical stability	ZnO nanorods, TiO_2_ thin films	[56]
Fractal Nanostructures	Surface enhancement, optical signal amplification	Increased active surface area	Fractal gold nanoarrays	[57]

**Table 3:** Comparison of intravascular and conventional biosensors.

Feature	Intravascular Biosensors	Subcutaneous/Wearable Biosensors	Refs.
Access to biomarkers	Direct and continuous access to blood plasma	Indirect via interstitial fluid; delayed correlation	[76,77]
Measurement lag	Minimal lag (seconds)	Significant delay (minutes) due to diffusion	[78,79]
Response time	Rapid sampling suitable for ICU/surgery settings	Slower response not optimal for acute care	[77]
Clinical relevance	Plasma-level accuracy, suitable for dynamic drug/metabolite monitoring	Moderate/correlated to interstitial changes	[79]
Biocompatibility requirements	Very high—must minimize clotting, inflammation, biofouling	Moderate level for skin contact	[77]
Thrombosis/infection risk	Elevated risk if coatings/materials are suboptimal	Lower risk, mainly surface exposure	[80]
Integration potential	Compatible with catheters, closed-loop pumps, stent-integrated systems	Primarily diagnostic, limited actuation capabilities	[81]
Maintenance/calibration	Challenging in vivo drift, difficult recalibration	Easier; patient-controlled recalibration possible	[80]

**Table 4:** Biomarkers and their diagnostic applications.

Biomarker	Diagnostic Applications	Detection Methods	Advantages	Refs.
Glucose	Diabetes monitoring	Electrochemical biosensors	Fast and accurate detection	[17,92]
Troponin	Detection of myocardial damage	Immunosensors	High specificity for the heart	[93]
C-reactive protein (CRP)	Diagnosing inflammation and infections	Biochemical tests, biosensors	Rapid inflammation detection	[94]
Genetic mutations (DNA/RNA)	Cancer diagnostics, genetic disorders	NGS, PCR, genomic analyses	Therapy personalization, early diagnostics	[95,96]
Hemoglobin detection	Fast and reliable blood test, tracking medical disorders, such as anemia	Biosensor grounded on metasurfaces	High sensitivity, achieving a peak value of 267 GHzRIU^−1^	[97]
Alanine aminotransferase (ALT), aspartate aminotransferase (AST)	Diagnosis of heart failure and liver injury, as well as various tissues in the organism	Working electrode altered with nanomaterials	Opportunity to monitor, among others, liver conditions	[98]
Alkaline phosphatase (ALP)	Detection of diseases of bone and hepatic dysfunction	Phosphorylated DNA probe	High sensitivity of detecting	[99]
α-Amylase	Detecting acute pancreatitis and psychological stress	Fluorescent biosensor arrays	Accurate determination of α-amylase concentrations in serum and saliva	[100]

**Table 5:** Examples of drugs administered with intravascular biosensors.

Drug	Disease/Condition	Type of Biosensor	Details/Outcomes	Refs.
Vancomycin	Severe bacterial infections	Fluorescence-based biosensor	Monitors drug levels in real time, reducing risks of nephrotoxicity and ototoxicity. Allows precise dosing adjustments.	[33]
Insulin	Diabetes mellitus	Electrochemical glucose biosensor	Continuous monitoring and real-time insulin delivery to maintain glucose control.	[119,120]
Chemotherapy drugs (e.g., doxorubicin)	Cancer	Electrochemical biosensor	This feedback-loop system enables precise, patient-specific dosing of drugs within narrow therapeutic windows.	[121]
Immunosuppressants (e.g., cyclosporine)	Transplant medicine	Optical biosensor	It combines the potential of microdialysis with an optical immunosensor in the therapeutic drug monitoring of immunosuppressants.	[122]
Propofol	Total intravenous anesthesia	Electrochemical measurement using biosensor-enabled catheter	This biosensor enables the detection of the propofol present in blood, and it is characterized by the accuracy, specificity, and high stability of the emitted signal.	[81]
Propofol and fentanyl	Anesthesia	Electrochemical sensor	Real-time monitoring of the concentrations of both propofol and fentanyl simultaneously throughout surgical operations using a dual-analyte microcatheter-based system.	[123]

## Data Availability

Data sharing is not applicable to this article.
